# Evaluation of a commercial liquid-chromatography high-resolution mass-spectrometry method for the determination of hepcidin-25

**DOI:** 10.11613/BM.2019.020701

**Published:** 2019-04-15

**Authors:** Dietmar Enko, Sieglinde Zelzer, Günter Fauler, Markus Herrmann

**Affiliations:** 1 Institute of Clinical Chemistry and Laboratory Medicine, General Hospital Steyr, Steyr, Austria; 2 Clinical Institute of Medical and Chemical Laboratory Diagnostics, Medical University of Graz, Graz, Austria

**Keywords:** hepcidin-25, preanalytical phase, protein biomarker, liquid-chromatography high-resolution mass-spectrometry

## Abstract

**Introduction:**

Reliable determination of hepcidin-25, a key regulator of iron metabolism, is important. This study aimed at evaluating the performance of the Hepcidin-25 Liquid Chromatography-Tandem Mass-Spectrometry (LC-MS/MS) Kit (Immundiagnostik AG, Bensheim, Germany) for quantification of the hepcidin-25 protein.

**Materials and methods:**

Precision, accuracy, linearity, and preanalytical requirements of the liquid-chromatography high-resolution mass-spectrometry (LC-HR-MS) method were evaluated. The imprecision and bias acceptance criteria were defined ≤ 15%. We investigated sample stability at room temperature (RT) and after repeated freeze and thaw cycles. Additionally, we assessed serum hepcidin-25 concentrations of 165 healthy adults referred for a medical check-up.

**Results:**

The hepcidin-25 LC-MS/MS assay was linear over the concentration range of 3 – 200 ng/mL. Within- and between-run precision ranged between 1.9 – 8.6% and 5.1 – 12.4%, respectively. The mean bias of the low and high control material was - 2.7% and 2.1%, respectively. At RT, serum samples were stable for 3 h (mean bias + 0.3%). After two and three freeze and thaw cycles, hepcidin-25 concentrations showed a bias of + 8.0 and + 20%, respectively. Of 165 healthy adults, 109 females had a significantly lower median of 8.42 (range: 1.00 – 60.10) ng/mL compared to 56 males with 15.76 (range: 1.50 – 60.50) ng/mL (P = 0.002).

**Conclusions:**

The hepcidin-25 LC-MS/MS kit shows a broad analytical range and meets the imprecision and bias acceptance criteria of ≤ 15%. Serum samples can be stored at RT for 3 h and resist up to two freeze and thaw cycles.

## Introduction

In recent years, hepcidin-25, an essential key regulator of human iron homeostasis, has gained substantial attention. This cysteine-rich acute-phase protein, which consists of 25 amino acids, is synthesized in the liver and excreted by the kidneys (*1*, *2*). The hepatic synthesis of hepcidin-25 is induced by iron loading or inflammation and inhibited by erythropoiesis (*3*, *4*).

Hepcidin-25 lowers circulating iron in the bloodstream by binding to and downregulating the cellular iron efflux channel ferroportin, which is highly expressed in duodenal enterocytes and macrophages of the reticuloendothelial system (*5*, *6*). Increased serum hepcidin-25 concentrations decrease the enteral iron absorption and the release of stored iron from macrophages and hepatocytes (*4*). Conversely, suppressed hepcidin-25 production enhances intestinal iron absorption and the ability of the reticuloendothelial system to export recycled iron from senescent erythrocytes (*4*, *7*).

As hepcidin-25 is a promising biomarker in the assessment of the human iron status, the quantitative analysis of this parameter is of great interest. In clinical practice, the establishment of various diagnostic tools (*i.e.* immunoassays, liquid chromatography-tandem mass-spectrometry (LC-MS/MS)) showed substantial differences in absolute hepcidin-25 concentrations and reproducibility of results between routine laboratories (*8*). Although no gold standard procedure for hepcidin-25 measurements has been defined yet, LC-MS/MS has been proposed to be more specific and sensitive compared to immunoassays (*8*-*10*).

The LC-MS/MS method is a powerful and valuable tool, which has become a widely used technique for quantitative determination of small molecules (*i.e.* steroid hormones) (*11*). High specificity, precision and flexibility, together with the potential of simultaneous determination of many different target compounds are the main advantages of this method (*12*). However, at present clinical laboratories rarely use LC-MS/MS for the quantitation of proteins in daily practice. Large molecule size and the complexity of the matrix are challenges for accurate quantification (*10*).

Beside the triple-quadrupole mass-spectrometry (QQQ-MS) with low resolution, there are also high-resolution (HR) instruments with ion trap MS available, which may include q-Orbitrap-MS (Q-Orbi-MS) and q-time-of-flight-MS (Q-TOF-MS). High-resolution MS is more applicable for analysis of intact peptides and proteins compared to quadrupole-instruments working in unit resolution (*13*, *14*). Furthermore, HR-MS instruments have the advantage that data can be acquired also in full-scan mode, allowing retrospective search for compounds, not initially targeted.

Currently, the Hepcidin-25 LC-MS/MS Kit (Immundiagnostik AG, Bensheim, Germany) and the Hepcidin-25 LC-MS/MS Kit (Li StarFish, Cernusco, Italy) are commercially available to facilitate the quantification of this protein biomarker via LC-MS/MS in clinical research. However, a thorough evaluation of the analytical performance by the use of an HR-MS instrumentation is recommended before these assays can be used in clinical routine. The aim of the present study was to evaluate the precision, accuracy, linearity, the limit of detection (LoD), and the limit of quantification (LoQ) of the Hepcidin-25 LC-MS/MS Kit from Immundiagnostik AG with a HR-MS method. Additionally, we performed a stability study evaluating sample storage at room temperature (RT), repeated freeze and thaw cycles, and the auto-sampler stability.

## Materials and methods

### Subjects

For the performance evaluation of the Hepcidin-25 LC-MS/MS Kit (Immundiagnostik AG, Bensheim, Germany), remaining blood samples (serum) from routine analysis of 165 ambulatory healthy adults, who were referred to the outpatient clinic of the Institute of Clinical Chemistry and Laboratory Medicine of the General Hospital Steyr (Steyr, Austria) for a medical check-up of the iron status, were used. The study period was from January to June 2017. A total number of 109 individuals (66%) were females and 56 (34%) were males. The median age was 43 (range: 15 – 90) years. All participants provided their written informed consent. They underwent blood sampling after an overnight fasting state (12h) in the morning between 08:00 and 10:00 a.m. Four mL VACUETTE® Z Serum Clot Activator tubes (Greiner Bio-one International GmbH, Kremsmünster, Austria) were used for blood draw from a peripheral vein. Serum samples were centrifuged at 1800xg for 10 minutes at RT and immediately analysed after blood draw. All 165 serum samples were used to evaluate and compare hepcidin-25 medians between females and males.

The study was approved by the Ethical Committee of the Johannes Kepler University Linz (Linz, Austria) and carried out in accordance with the current version of the Declaration of Helsinki.

### Materials

#### Chemicals

The Hepcidin-25 LC-MS/MS Tuning Kit (Immundiagnostik AG, Bensheim, Germany) was used for optimization of LC-HR-MS ionization settings. This Tuning Kit consists of a highly pure hepcidin-25 and the internal standard (IS), Calcitonin Gene related Peptide human, each with a concentration of 1 µg/mL. For serum hepcidin-25 measurements, the Hepcidin-25 LC-MS/MS assay was purchased from Immundiagnostik AG. All further reagents and solvents of the kits are described in the manuals of the manufacturer in detail. Oasis^®^ hydrophilic-lipophilic-balanced (HLB), 1cc (10 mg) cartridges (Waters, Eschborn, Germany) and foetal bovine serum (Sigma-Aldrich, Vienna, Austria) were used.

#### Instrumentation and conditions

An ultra-high-pressure liquid-chromatography (UHPLC) Accela 1250 pump, a column oven (MayLab, Vienna, Austria) and an auto-sampler Accela Open AS were coupled on a Q Exactive hybrid Q-Orbitrap-MS (ThermoFisher Scientific, San Jose, California). The LC-MS instrument control was performed using Xcalibur^TM^ software version 2.2. (ThermoFisher Scientific, San Jose, California). For chromatography, a XSelect charged surface hybrid (CSH) C18 column (130Å, 3.5 µm, 2.1 mm x 50 mm; Waters, Eschborn, Germany) and a gradient of mobile phase A and B (Immunodiagnostics AG, Bensheim Germany) were used for separation and elution. Gradient settings for eluents A/B (in %, v/v) were, 90/10 (0 min), 90/10 (1.50 min), 5/95 (7 min), 5/95 (8 min), 90/10 (9 min), 90/10 (10 min; re-equilibration start). Flow rate was 400 μL/min. Ionization in positive mode was performed with a heated electrospray ionization (ESI) ion source. In brief: sheath gas flow rate 35 mL/min, auxiliary gas flow rate 10 mL/min, sweep gas flow rate 0 mL/min, spray voltage 4.00 kV, capillary temperature 350 °C, S-lens radio frequency (RF) level 90, and auxiliary gas heater temperature 150 °C. Positive ion full scan mode was set between mass to charge ratio (m/z) = 740 to m/z = 950, resolution was 70,000 (specified at m/z = 200).

**Figure 1:**
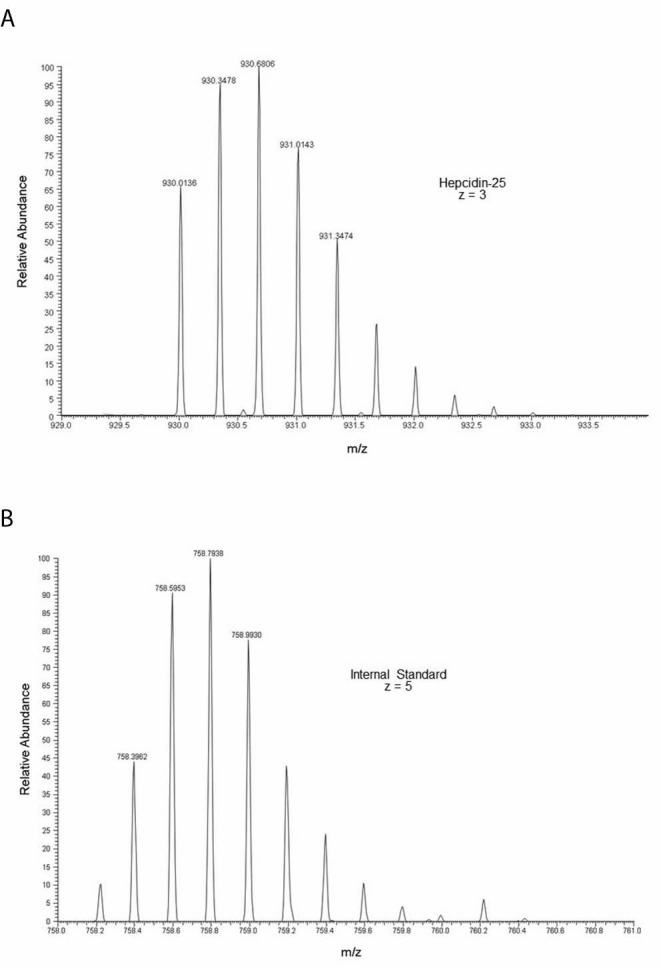
Isotopic distribution and accurate mass extraction of the molecular mass from hepcidin-25 and the internal standard, Calcitonin Gene related Peptide human. (A) Relative isotopic distribution of hepcidin-25. The threefold charged ion [M+3H]^3+^ revealed highest intensity. This and the next three isotopic peaks were used for quantification. (B) Relative isotopic distribution of the internal standard. Most intense ion was the fivefold charged ion [M+5H]^5+^. This and the next three isotopic peaks were used for quantification. m/z – mass to charge ratio.

The isotopic abundance and the accurate mass extraction of hepcidin-25 and the IS are shown in Figure 1 (A and B). For isotopic distribution of hepcidin-25, the most abundant peak was the threefold charged ion [M+3H]^3+^, revealed as the highest intensity (A). This and the next three isotopic peaks were used for post-acquisition data processing of hepcidin-25. For the IS determination, the fivefold charged ion [M+5H]^5+^, which showed the highest intensity, together with the area of two further isotopic peaks were used (B). The deviations of the m/z of target ions from their theoretical masses were within the region of 6 ppm. Peak area ratios from hepcidin-25 versus the IS were calculated and used to construct the calibration curves (1/X˄2 weighting). Mass calibration of the instrument was carried out at least every third day.

### Methods

#### Preparation of calibration

Instead of the two-point calibration, as recommended by the manufacturer, an in-house calibration curve was prepared with analyte-free foetal bovine serum, which was spiked with hepcidin-25 (from the Tunning Kit), obtaining seven different concentrations (3.13, 6.25, 12.50, 25, 50, 100 and 200 ng/mL). Two- and seven-point calibration results were compared to prove that seven-point calibration yields comparable results.

#### Sample preparation

Oasis^®^ HLB 1cc (10 mg) cartridges (Waters, Eschborn, Germany) were conditioned by consecutive rinsing with methanol (200 μL) and deionized water (200 μL). 200 µL serum, together with 100 µL IS-solution, were loaded on the cartridges under vacuum followed by three washing steps using the wash solution (each with 200 µL), provided by the manufacturer. Analytes were eluted with the elution solution (100µL) from the kit and diluted equally (v/v, 60µL/60µL) with the wash solution 1. Subsequently 50 µL were injected onto the LC-HR-MS system.

#### Assay evaluation

Evaluation of the Hepcidin-25 LC-MS/MS Kit was performed according to the guidelines of the Clinical and Laboratory Standards Institute (CLSI) (*15*-*17*).

Precision measurements and recovery tests were assessed with seven calibrators with the expected hepcidin-25 concentrations of 3.13, 6.25, 12.50, 25, 50, 100 and 200 ng/mL (Table 1). Until analysis, the calibrators were stored at -20 °C. The within- and between-run precisions were assessed by replicate analyses (N = 5) of seven hepcidin-25 concentrations (3.13, 6.25, 12.50, 25, 50, 100 and 200 ng/mL) on the same day and on five consecutive days (*15*). According to the literature, the precision goal for each concentration was not to exceed 15% of the coefficient of variation (CV) (*18*).

**Table 1:** Precision studies for the hepcidin-25 liquid-chromatography high-resolution mass-spectrometry method

**Concentration of** **hepcidin-25 calibrator (ng/mL)**	**Within-run precision**		**Between-run precision**		**Recovery (%)**
	**Mean ± SD**	**CV (%)**	**Mean ± SD**	**CV (%)**	
1 (3.13)	3.30 ± 0.14	4.2	3.28 ± 0.10	3.1	106.6
2 (6.25)	6.60 ± 0.01	1.9	6.02 ± 0.43	7.2	103.2
3 (12.5)	10.90 ± 0.03	2.7	10.25 ± 1.14	11.1	88.0
4 (25)	25.80 ± 0.16	6.0	28.36 ± 3.53	12.4	94.4
5 (50)	49.40 ± 0.42	8.6	47.01 ± 4.61	9.8	95.6
6 (100)	102.30 ± 0.25	2.4	103.77 ± 5.28	5.1	100.2
7 (200)	205.70 ± 1.64	8.0	201.51 ± 18.97	7.0	98.8
SD - standard deviation. CV - coefficient of variation. The acceptance criteria for the precision studies were ≤ 15% of the CV. The average recovery was 98.1%.					

The accuracy was assessed as the difference between the result of the mean value of six measurements of the low and high control material provided by the manufacturer (Immundiagnostik AG, Bensheim, Germany) compared to its “true” value (*17*). According to the literature, the acceptance criteria for the accuracy were defined ≤ 15% (*18*).

The LoD was defined as the lowest concentration, which showed a signal of at least three times higher than the average background noise of an unspiked blank (*19*). For the determination of LoD, the lowest calibrator (3.13 ng/mL) was added by decreasing concentrations to the blank matrix.

The LoQ was defined as the lowest concentration that can be determined with an acceptable level of repeatability precision (< 10%) (*19*). The LoQ was performed by measuring the calibrator with lowest concentration (3.13 ng/mL) at five consecutive days.

The manufacturer’s claim for within- and between-run imprecisions were 2.6 and 3.8 – 7.3%, respectively. The LoD was quoted 1 ng/mL (*20*).

#### Analyte stability measurements

To investigate analyte stability, three serum samples were used to prepare a serum pool of 5 mL. This serum pool was divided in 20 aliquots (250 µL each), which were stored at RT. Three aliquots were measured at 0 and 3 h, and after 4 and 7 days. The RT in the laboratory is constant 25 °C and monitored by continuous record of the air conditioner.

A second serum pool of 5 mL was prepared with three other serum samples. This pool was also divided in 20 aliquots (250 µL each), which were stored at -20 °C. On days 1, 2 and 7, all aliquots were thawed, three of them were assayed and the rest again deep-frozen at -20 °C. A Kirsch MED-340 freezer (Kirsch, Offenburg, Germany) was used for -20 °C sample storage. The continuous record of the temperature ensures a high-quality monitoring. The specific concentrations for each time point of the three measured aliquots of both serum pools were calculated as arithmetic means.

The three aliquots of serum analyte stability on the auto-sampler tray (4 °C) was investigated with calibrator 3 and 4. Both calibrators were measured on days 1 – 4.

### Statistical analysis

Descriptive statistics was used to summarize and present the study results. The distribution of the hepcidin-25 measurements was calculated with the Kolmogorov-Smirnov test. The exact Mann-Whitney U-test was used for subgroup comparison. A P-value < 0.05 was considered statistically significant. Statistical tests were performed with the Analyse-it® software version 4.92 (Analyse-it Software, Ltd., Leeds, United Kingdom). The formulas for bias calculations were as follows: absolute bias (ng/mL) = measured concentration – expected concentration and measured concentration – initial concentration (in terms of stability measurements); mean bias (%) = measured value – expected value/expected value x 100.

## Results

### Analytical performance of the hepcidin-25 assay

**Figure 2:**
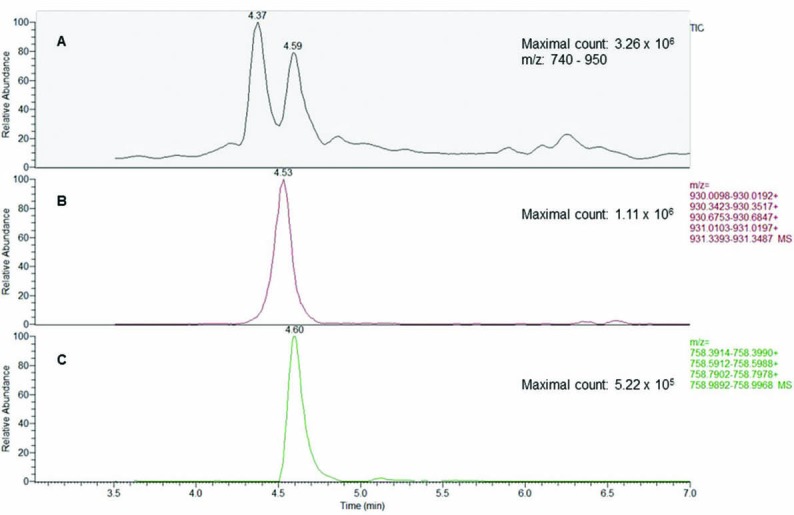
Representative chromatogram of high-resolution mass-spectrometry for the determination of hepcidin-25 concentration. (A) Total ion chromatogram. (B) Hepcidin-25 detected with a retention time at 4.54 min. (C) Internal standard (Calcitonin Gene related Peptide human) with a retention time at 4.58 min. m/z – mass to charge ratio.

Figure 2 (A-C) shows a representative chromatogram of the high-resolution technique of hepcidin-25 and of IS. The commercial Hepcidin-25 LC-MS/MS Kit was linear over the concentration range of 3 – 200 ng/mL. The coefficient of determination (r^2^) was 0.9898. LoD was 1 ng/mL and LoQ was 3 ng/mL respectively.

The results of the precision studies and the recovery of the LC-HR-MS method are shown in Table 1. Within-run CVs varied between 1.9 - 8.6% and between-run CVs ranged between 5.1 - 12.4% and were within the acceptance criteria of ≤ 15%. Observed recovery was between 88 – 107%.

The accuracy studies were within the acceptance criteria of ≤ 15% and showed an absolute and mean bias of - 1.25 ng/mL and - 2.7%, respectively, for the low control material (“true” *vs.* measured value: 45.50 *vs.* 44.25 ng/mL) and an absolute and mean bias of 2.28 ng/mL and 2.1%, respectively, for the high control material (“true” *vs.* measured value: 110.50 *vs.* 112.78 ng/mL).

Results of compared two-point and seven-point calibrations are shown in Table 2. The mean absolute and relative bias were - 0.8 ng/mL and - 11.3%, respectively.

**Table 2:** Hepcidin-25 values compared with two-point and seven-point calibrations

**Sample number**	**Hepcidin-25 (ng/mL) measured** **with two-point calibration**	**Hepcidin-25 (ng/mL) measured** **with seven-point calibration**	**Difference** **(ng/mL)**
**1**	3.8	2.5	- 1.3
**2**	7.4	6.7	- 0.7
**3**	9.2	8.0	- 1.2
**4**	6.1	5.3	- 0.8
**5**	2.6	2.4	- 0.2
**6**	26.2	24.6	- 1.6
**7**	1.5	1.7	+ 0.2
**8**	3.7	2.3	- 1.4
**9**	4.8	3.9	- 0.9
**10**	3.7	2.9	- 0.8
**11**	4.2	2.5	- 1.7
**12**	12.7	11.9	- 0.8
**13**	1.5	1.3	- 0.2
**14**	13.7	12.6	- 1.1
**15**	5.5	4.8	- 0.7
**16**	0.9	0.7	- 0.2
**17**	12.9	12.6	- 0.3
**18**	3.7	2.4	- 1.3
**19**	8.5	7.8	- 0.7
**20**	9.2	8.6	- 0.6
The mean absolute and relative bias between two-point and seven-point calibration was - 0.8 ng/mL and - 11.3% (bias acceptance criteria ≤ 15%), respectively.			

### Pre-analytical analyses

As shown in Table 3, serum samples were stable at RT for at least 3 h. The mean difference of repeated measurements within three aliquots of serum pool 1 after 3 h was + 0.3%. Mean hepcidin-25 concentrations decreased with - 49, - 68 and - 79% after 24 h, 4 days, and 7 days, respectively. Freeze and thaw cycle experiments demonstrated a mean hepcidin-25 concentration increase of + 1.4, + 8.0 and + 20% after specimens were thawed, analysed and again deep-frozen on day 1, day 2 and day 7, respectively.

**Table 3:** Hepcidin-25 stability measurements after room temperature storage and freeze and thaw cycle experiments

**Serum pool 1**					
**Hepcidin -25**	**Basal**	**RT 3 h**	**RT 24 h**	**RT 4 days**	**RT 7 days**
Mean ± SD (ng/mL)	13.68	13.72 ± 0.03	7.02 ± 0.48	4.32 ± 0.09	2.85 ± 0.03
Difference (%)	/	+ 0.3	- 49	- 68	- 79
CV (%)	/	0.21	6.86	2.12	1.13
**Serum pool 2**					
**Hepcidin-25**	**Basal**	**1 freeze and thaw cycle**	**2 freeze and thaw cycles**	**3 freeze and thaw cycles**	
Mean ± SD (ng/mL)	7.39	7.49 ± 0.99	8.09 ± 1.09	8.99 ± 0.73	
Difference (%)	/	+ 1.4	+ 8.0	+ 20	
CV (%)	/	13.4	13.5	8.1	
SD - standard deviation. CV - coefficient of variation. Aliquots of serum pool 1 were measured after 3 h, 24 h, 4 days and 7 days storage at room temperature (RT). The mean decrease of hepcidin-25 concentrations after 7 days was - 79%. Aliquots of serum pool 2 were stored at - 20°C. The mean hepcidin-25 concentration after 1, 2 and 3 freeze and thaw cycles were + 1.4, + 8.0 and + 20% (acceptance criteria ≤ 15%), respectively.					

**Figure 3:**
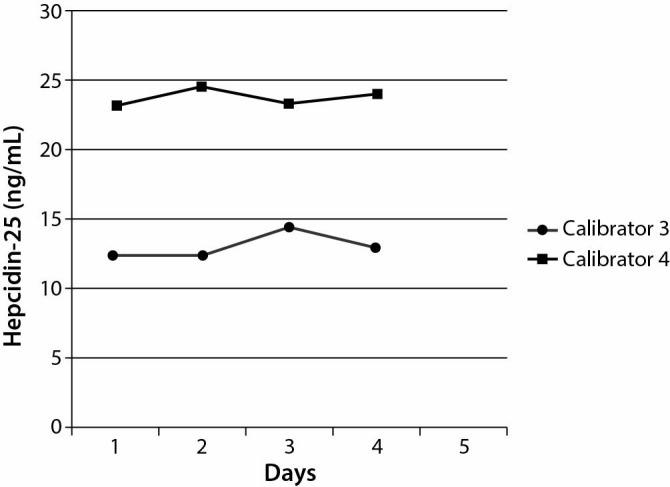
Auto-sampler stability measurements (4 °C). After 4 days, the hepcidin-25 measurements with calibrator 3 (12.5 ng/mL) and 4 (25 ng/mL) were stable. The coefficients of variation (CVs) for calibrator 3 and 4 were 8.6 and 2.6% (acceptance criteria ≤ 15%), respectively.

Auto-sampler stability measurements (4 °C) with calibrator 3 (12.50 ng/mL) and 4 (25.0 ng/mL) are shown in Figure 3. After 4 days, the hepcidin-25 measurements were stable. The CVs for calibrator 3 and 4 were 8.6% (10.80 ± 0.92 ng/mL) and 2.6% (23.80 ± 0.62 ng/mL), respectively.

### Hepcidin-25 serum concentrations

Hepcidin-25 measurements of all included individuals (N = 165) showed a median value of 10.80 (range: 1.0 – 60.50) ng/mL. Females (N = 109) had a significantly lower median of 8.42 (range: 1.0 – 60.10) ng/mL compared to males (N = 56) with 15.76 (range: 1.50 – 60.50) ng/mL (P = 0.002), respectively. Sex-related hepcidin-25 serum medians stratified by 10-year groups were lower in females than males in all decades of life with the exception of the age groups 60 – 69 years and < 20 years, in which women showed higher medians compared to men (data not shown).

## Discussion

In the present study, the within- and between-run imprecisions of the Hepcidin-25 LC-MS/MS Kit from Immundiagnostik AG applied on a HR-MS instrumentation varied between 1.9 - 8.6% and 5.1 - 12.4%. The LoD was 1 ng/mL. These results were in line with the manufacturer’s specifications quoted for within- and between-run imprecisions and LoD (*20*). The accuracy (bias) studies were ≤ 15%, which conform with the acceptance criteria of the Food and Drug Administration (FDA) Guidelines for Bioanalytical Method Validation (*18*).

Here, higher CVs were observed for higher and lower CVs for lower analyte concentrations. The authors themselves cannot fully explain this phenomenon but to our experience, the performance of LC-MS/MS methods is often so that at higher analyte concentrations, the ionisation leads to more imprecision. A second point may be the fact that we did not use an isotopically labelled IS in our study.

Beside the two-point calibration curve proposed by the manufacturer, we additionally fitted a calibration curve with seven in-house calibration standards. Such a multi-point calibration is proposed in the validation recommendations for LC-MS/MS methods (*18*). Present data show the equivalence of the two calibrations (mean bias ≤ 15%). Our laboratory prefers the seven-point calibration curve because the lowest calibrator of the manufacturer was 22.1 ng/mL and the determined LoQ was 3.1 ng/mL.

LC-HR-MS has been shown as an accurate and reliable technique for the quantitative determination of small molecules in clinical routine, for example in determination of bile acids (*21*). Performing Q Exactive MS instruments with Orbitrap-technology, proteins up to 30 kDa can be isotopically resolved by the use of high resolution. Accurate quantification with significantly increased sensitivity can be achieved by summarizing the area of all resolved isotope-peaks of a particular ionization status. In the current literature, only two publications describe the determination of hepcidin-25 performed with LC-HR-MS (*22*, *23*). To the best of our knowledge, this is the first report evaluating the commercially available Hepcidin-25 LC-MS/MS Kit with this high-resolution technique.

Within the last years, the diagnostic use of LC-MS/MS methods and immunoassays for human serum hepcidin-25 measurements increased rapidly (*2*, *24*-*26*). Earlier studies, which report the development of quantitative in-house hepcidin-25 LC-MS/MS methods, used synthetic human hepcidin-25 from Peptide Institute (Osaka, Japan) for standard curve calculations (*24*-*26*). In comparison, we used the hepcidin-25 of the Tunning Kit from Immundiagnostik AG (Bensheim, Germany). Various sources of synthetic hepcidin-25 and different protocols for sample preparation and chromatographic separation could be possible reasons for differences observed between LC-MS/MS methods (*26*). In addition, pre-analytical factors, sample storage and analyte stability must be considered in order to obtain reproducible and comparable results (*27*). Moreover, circulating hepcidin-25 concentrations underlie a circadian rhythm with lowest levels in the morning and highest values in the afternoon (*28*).

Herein, we performed blood sampling in a fasting state in the early morning and studied preanalytical stability measurements. At ambient RT, serum samples were stable for up to 3 h. The mean decrease of hepcidin-25 concentration after 24 h was - 49%. These data indicate that delays in transportation, aliquoting or measuring hepcidin-25 blood samples at RT should be avoided (*29*). In comparison, a previous study reported hepcidin-25 serum concentrations to be stable at RT for one day (*27*). Recently, Handley *et al.* showed serum hepcidin-25 measurements to be stable for at least up to three weeks (*23*). The authors presumed that protein LoBind tubes, which are especially designed to minimize protein absorption, had contributed to this extraordinary hepcidin-25 stability at RT (*23*).

In this work, hepcidin-25 measurements were stable after two freeze and thaw cycles. Previous reports showed analyte stabilities for at least three and five repeated analyses after freezing (- 20°C) and thawing (RT) of hepcidin-25 serum samples (*23*, *27*). All these data are indicative for the prevention of repeated freeze and thaw cycles in laboratories, which handle hepcidin-25 measurements in a patch workflow.

Herein, females showed significantly lower hepcidin-25 serum concentrations compared to males (P = 0.002). This finding is in line with previous published studies, which reported that serum hepcidin-25 concentrations are substantially higher in men than in women (*27*, *30*). It is believed that iron loss during menstruation explains this gender differences (*27*, *30*).

Several limitations of this study must be mentioned. For precision and analyte stability testing calibration samples were used because it was difficult to get samples of patients. Reference ranges were not calculated because data on subjects’ disorders or therapy, which are necessary for this determination, were not included. Hepcidin-25 stability measurements were not performed with pathological analyte concentration and determinations at RT were performed within the first 24 h, only.

In conclusion, the Hepcidin-25 LC-MS/MS Kit from Immundiagnostik AG shows a broad analytical range and meets the imprecision and bias acceptance criteria of ≤ 15%. Serum samples can be stored at RT for 3 h and resist up to two freeze and thaw cycles. These data are indicative for a reliable and robust diagnostic method for clinical practice.

## References

[1] Galesloot TE Vermeulen SH Geurts-Moespot AJ Klaver SM Kroot JJ van Tienoven D Serum hepcidin: reference ranges and biochemical correlates in the general population. Blood. 2011;117:e218–25. 10.1182/blood-2011-02-337907 21527524

[2] Enko D Wagner H Kriegshäuser G Kimbacher C Stolba R Worf E Hepcidin-25 vs. conventional clinical biomarkers in the diagnosis of functional iron deficiency. Eur J Haematol. 2015;95:507–13. 10.1111/ejh.12523 25598480

[3] Nemeth E Valore EV Territo M Schiller G Lichtenstein A Ganz T Hepcidin, a putative mediator of anemia of inflammation, is a type II acute-phase protein. Blood. 2003;101:2461–3. 10.1182/blood-2002-10-3235 12433676

[4] Young B Zaritsky J Hepcidin for clinicians. Clin J Am Soc Nephrol. 2009;4:1384–7. 10.2215/CJN.02190309 19556376

[5] Zhao N Zhang AS Enns CA Iron regulation by hepcidin. J Clin Invest. 2013;123:2337–43. 10.1172/JCI67225 23722909 PMC3668831

[6] Nemeth E Tuttle MS Powelson J Vaughn MB Donovan A Ward DM Hepcidin regulates cellular iron efflux by binding to ferroportin and inducing its internalization. Science. 2004;306:2090–3. 10.1126/science.1104742 15514116

[7] Ganz T Hepcidin and iron regulation, 10 years later. Blood. 2011;117:4425–33. 10.1182/blood-2011-01-258467 21346250 PMC3099567

[8] van der Vorm LN Hendriks JC Laarakkers CM Klaver S Armitage AE Bamberg A Toward worldwide hepcidin assay harmonization: identification of a commutable secondary reference material. Clin Chem. 2016;62:993–1001. 10.1373/clinchem.2016.256768 27173010

[9] Keevil BG LC-MS/MS analysis of steroids in the clinical laboratory. Clin Biochem. 2016;49:989–97. 10.1016/j.clinbiochem.2016.04.009 27131495

[10] Schmitz EMH Leijten NM van Dongen JLJ Broeren MAC Milroy LG Brunsveld L Optimizing charge state distribution is a prerequisite for accurate protein biomarker quantification with LC-MS/MS, as illustrated by hepcidin measurement. Clin Chem Lab Med. 2018;56:1490–7. 10.1515/cclm-2018-0013 29777607

[11] Ketha SS Singh RJ Ketha H Role of mass spectrometry in clinical endocrinology. Endocrinol Metab Clin North Am. 2017;46:593–613. 10.1016/j.ecl.2017.04.001 28760228

[12] Leung KS Fong BM LC-MS/MS in the routine clinical laboratory: has its time come? Anal Bioanal Chem. 2014;406:2289–301. 10.1007/s00216-013-7542-5 24337187

[13] Grund B Marvin L Rochat B Quantitative performance of a quadrupole-orbitrap-MS in targeted LC-MS determinations of small molecules. J Pharm Biomed Anal. 2016;124:48–56. 10.1016/j.jpba.2016.02.025 26928213

[14] Rochat B From targeted quantification to untargeted metabolomics: Why LC-high-resolution-MS will become a key instrument in clinical labs. Trends Analyt Chem. 2016;84:151–64. 10.1016/j.trac.2016.02.009

[15] Clinical and Laboratory Standards Institute (CLSI). Evaluation of Precision of Quantitative Measurement Procedures; Approved Guideline – Third Edition. CLSI document EP05-A3. Wayne, PA, USA, 2014.

[16] Clinical and Laboratory Standards Institute (CLSI). Evaluation of the Linearity of Quantitative Measurement Procedures: A Statistical Approach; Approved Guideline. CLSI document. EP06-A. Wayne, PA, USA, 2003.

[17] Clinical and Laboratory Standards Institute (CLSI). Measurement Procedure Comparison and Bias Estimation Using Patient Samples; Approved Guideline – Third Edition. CLSI document EP-09-A3. Wayne, PA, USA, 2013.

[18] U.S. Department of Health and Human Services, Food and Drug Administration (FDA), Center for Drug Evaluation and Research (CDER), Center for Veterinary Medicine (CVM). Bioanalytical Method Validation – Guidance for Industry. Available at: https://www.fda.gov/downloads/Drugs/Guidances/ucm070107.pdf. Accessed June 15th 2018.

[19] Thompson M Ellison SLR Wood R Harmonized guidelines for single laboratory validation of methods of analysis. Pure Appl Chem. 2002;74:835–55. 10.1351/pac200274050835

[20] Hepcidin 25 LC-MS/MS Kit – For the determination of hepcidin in serum. Immundiagnostik AG. Bensheim, Germany, 2014.

[21] Amplatz B Zöhrer E Haas C Schäffer M Stojakovic T Jahnel J Bile acid preparation and comprehensive analysis by high performance liquid chromatography-high-resolution mass spectrometry. Clin Chim Acta. 2017;464:85–92. 10.1016/j.cca.2016.11.014 27838249

[22] Rochat B Peduzzi D McMullen J Favre A Kottelat E Favrat B Validation of hepcidin quantification in plasma using LC-HRMS and discovery of a new hepcidin isoform. Bioanalysis. 2013;5:2509–20. 10.4155/bio.13.225 24138624

[23] Handley S Couchman L Sharp P Macdougall I Moniz C Measurement of hepcidin isoforms in human serum by liquid chromatography with high resolution mass spectrometry. Bioanalysis. 2017;9:541–53. 10.4155/bio-2016-0286 28229619

[24] Lefebvre T Dessendier N Houamel D Ialy-Radio N Kannengiesser C Manceau H LC-MS/MS method for hepcidin-25 measurement in human and mouse serum: clinical and research implications in iron disorders. Clin Chem Lab Med. 2015;53:1557–67. 10.1515/cclm-2014-1093 25781546

[25] Swensen AC Finnell JG Matias C Gross AJ Prince JT Watt RK Whole blood and urine bioactive hepcidin-25 determination using liquid chromatography mass spectrometry. Anal Biochem. 2017;517:23–30. 10.1016/j.ab.2016.10.023 27794422

[26] Abbas IM Hoffmann H Montes-Bayón M Weller MG Improved LC-MS/MS method for the quantification of hepcidin-25 in clinical samples. Anal Bioanal Chem. 2018;410:3835–46. 10.1007/s00216-018-1056-0 29666914

[27] Itkonen O Parkkinen J Stenman UH Hämäläinen E Preanalytical factors and reference intervals for serum hepcidin LC-MS/MS method. Clin Chim Acta. 2012;413:696–701. 10.1016/j.cca.2011.12.015 22222553

[28] Manolov V Velizarova M Atanasova B Vasilev V Lambreva L Tzatchev K Preanalysis in serum hepcidin measurement. Clin Lab. 2015;61:647–9. 10.7754/Clin.Lab.2014.141114 26118202

[29] Laarakkers CM Wiegerinck ET Klaver S Kolodziejczyk M Gille H Hohlbaum AM Improved mass spectrometry assay for plasma hepcidin: detection and characterization of a novel hepcidin isoform. PLoS One. 2013;8:e75518. 10.1371/journal.pone.0075518 24124495 PMC3790851

[30] Wolff F Deleers M Melot C Gulbis B Cotton F Hepcidin-25: measurement by LC-MS/MS in serum and urine, reference ranges and urinary fractional excretion. Clin Chim Acta. 2013;423:99–104. 10.1016/j.cca.2013.04.021 23643854

